# Comparative Physiology

**Published:** 1836

**Authors:** 


### REVIEWS AND BIBLIOGRAPHICAL NOTICES.

### Art. VI.—Comparative Physiology.

*A Systematic Treatise on Comparative Physiology Introductory to the Physiology of Man.*—Translated, with notes from the German of Frederick Tiedemann, Professor of Anatomy and Physiology in the Heidleberg University. By James Manby Gully, M. D., and J. Hunter Lane, M. D., etc. London, John Churchill, p. 431. 8 vo.

Few writers have done more for physiology than Tiedemann. The work before us appears to embrace the principles, and doctrines of his lectures, on the subject, delivered in the University of Heidelberg. It is dedicated to his pupils, by a short preface, in which the author informs them that he has enlarged upon the principal subjects touched on in his previous lectures. He commences his inquiries by instituting a comparison between the phenomena of organic and inorganic bodies. This is immediately followed by similar inquiries into the properties and circumstances which distinguish the two great classes of living bodies—animal and vegetable. After this subject is examined, in all its bearings, our author ascends still higher in the scale of life, and compares the formation, powers, and functions of man with the inferior animals, and points out the particular station of each in the scale of organization. Thus the powers and functions of the entire organic world, with the peculiarities of each class, are made known to the student. Each step he takes in the study of physiology in this way, opens to him new views of his subject. The study is also continually increasing in interest until the last, when the beauty and order of creation, is spread out before him in all its grandeur.

We have long thought that this was the best mode of studying physiology, and hence we sometime since adopted this plan, with interest to ourselves at least, in a course of lectures, delivered on the subject, in this city, without being aware that we were following the arrangement of the Heidelberg Professor. The truth is that writers on physiology have given their attention almost entirely to the support of favorite theories, without collecting, comparing or arranging the facts necessary for the development of truth. In many instances we can only ascertain the functions of various organs by comparing different animals, plants and even minerals with each other. If we find a multiplication of organs as we rise in the scale of life, we must also find a greater variety of functions, and vice versa. The work of Tiedemann may strictly be termed a system of comparative physiology in which the uses of the organs in one animal are determined by comparing it with others where such organs do not exist. And this at last, we again repeat, is the only correct mode of studying physiology. It is true that experiment aids us much in our inquiry, but were we to rely entirely upon it in our investigations, we should frequently be led astray, for how often do different persons arrive at opposite conclusions from the same experiments? Legallois and Philip, both men of talents and research, presented the world with different views of the same subject, drawn from similar experiments on animals.

After enumerating a great variety of ways in which organic bodies differ from inorganic, our author holds the following language concerning the doctrines of spontaneous generation.

"Regarding the origin of organic combinations, experience teaches us that they are only produced by the manifestations of activity of living bodies already existing. Albumen, gelatin, mucus, gluten, starch, gum, sugar, etc., do not form spontaneously, by the union of elements, or binary compounds, according to the laws of chemical affinity, but only by the manifestations of activity of organic bodies already possessed of life. Organized beings are produced by their fellow-beings, or owe their origin to the matter of organized bodies in a state of decomposition. The production of organic combinations in these beings, takes the name of assimilation and nutrition, whilst the procreation of beings themselves is called generation. On the other hand, inorganic combinations and bodies never originate but from the remains of other more ancient bodies, fallen into dissolution, and the materials of which, under certain circumstances, reunite, to produce them, according to the laws of chemical and mechanical attraction alone.

Even the most simple animal and vegetable forms, the infusoria, the green matter of Priestley, the confervæ, mouldiness, etc., in what is called spontaneous generation, proceed, according to the observations and experiments undertaken by Needham, Priestley, Ingenhouz, Monti, Wrisberg, Muller, G. R. Treviranus, etc., not from inorganic matters, but from organic bodies and combinations passed into putrefaction or fermentation. It is true, that J. B. Fray asserts, he has seen infusoria animalculæ developed in pure water. Gruithuisen says, also, that he saw generated, in an infusion of granite, of chalk, and of marble, a gelatinous membrane, in which, after sometime, movements were manifested, and ended by the formation of infusoria, of monads, and globular animalculæ. But it is very probable that the bodies subjected to experiment, or the water employed in the infusion, already contained organic matter, though in a very small quantity, for other naturalists have not observed the formation of living bodies in infusions of purely inorganic ones."

Although our author appears, from the remarks just quoted, to disbelieve the hypothesis of equivocal generation, still it would seem from his future remarks, that he had embraced the doctrines of many of the French and German philosophers, in regard to the origin of the different species of organized beings. In order to give our readers some ideas of the various doctrines of the school to which he belongs, we quote the following:

"The principal result of the comparisons made between the composition of organic bodies and those of inorganic bodies, which comparisons are founded on observations and researches in the chemical composition of these bodies hitherto pursued, is, that the former have peculiar matters, which we call organic, for their basis. The changes of composition which take place in bodies endued with life, are not simply the effects of affinities similar to those observed in burnt or inorganic bodies; they are the effects of affinities and forces of a special nature. Organic matters are the only ones which exhibit, and for the greater period of time in a particular state of aggregation and form called organization, the manifestations of activity which we designate by the name of life. They are accumulated at one time in bodies actually living, and life is manifested in them; at other times they are exterior to living bodies, mixed with inorganic matters, and then only capable of living. In this latter state they may return to the domain of living bodies, and into the tide of life, either in the shape of aliments, or, in a direct manner, by the aid of certain circumstances, as happens in spontaneous generation. Purely chemical affinities, or the action of simple chemical forces, appear, in the present state of our planet, to produce no organic combination or matter, such as albumen, gelatin, starch, gluten, etc.; at least we possess no facts which go to support the contrary opinion. None but organic bodies themselves are capable of introducing inorganic matters into organic combinations, of which respiration in particular and the nutrition of plants are examples.

If we extend our researches still further, a question presents itself, namely, how organic matters, their different combinations and living bodies, are formed in our planet? The solution of this problem passes the limit of our experience. Should we, however, wish to hazard an answer to it, we fall into the waste of conjecture, and are forced to erect hypotheses, which are but probable, and not at all certain. We suppose that organized bodies have existed in our planet from its commencement; or else we admit that organic matters and living bodies have been produced, under certain circumstances, together with the elements and inorganic matters, by the action of general physical causes; or, lastly, we conjecture that the substance of living bodies was primitively contained in water, as primitive organic matter, having the property of taking on the organic form, that it gave origin to organic bodies of very simple and varied kinds in consequence of circumstances, and that these bodies have passed successively to more complicated forms, until at length the generative organs and their manifestations of activity having appeared in them, they were endowed with the faculty of preserving themselves in a continuous manner, by means of generation, as separate species.

Geology is opposed to the first hypothesis of the existence, in our planet, of living bodies from the first moments of its creation. Fossils are found only in the exterior crust, that is to say, in the superficial layers of the earth the formation which is most recent, whilst there are none at all in the primitive earths. Consequently there was a time when no living being existed on the globe. Even supposing we admitted this hypothesis, we should still leave untouched the question, how living bodies were formed, inasmuch as we could say nothing concerning the mode of origin of our planet and of the bodies which constitute it. In reference to this question, it matters little whether we declare for Vulcanism or Neptunism, since the geologists are under the necessity of leaving the origin of fire and water without explanation, and the biologist is still less able to pronounce any opinion on that of living bodies.

The difficulties which occur in the second hypothesis, of the dependence of the production or organic matters, and living bodies on the action of general physical forces, are, that we are actually in want of facts which would authorize us to conclude analogically that organic matters and living bodies can proceed from inorganic matters, never having observed any thing similar, at least up to this day. Far from this being the case, living bodies are unable to produce, with inorganic substances, the greater number of the materials which enter into their composition, and for such end they require the matter of other organic bodies, which they introduce into themselves. Plants are nourished principally by the remains of dead vegetables or animals: animals likewise preserve their existence by means of vegetables, and even of other animals.

The most probable hypothesis is the third, viz: that the substance of organic bodies existed primitively in water, as matter of a particular kind, and that it was there endowed with the plastic faculty, that is to say, with the power of acquiring, by degrees, different simple forms of living bodies, with the concurrence of the general influences of light, heat, and perhaps also of electricity, etc., and of then passing from the simple forms to other more complicated, varying in proportion to the modification occurring in the external influences until the point when each species acquired duration by the production and manifestation of activity of the genital organs. Although we cannot here also answer the question, whence came the water and the organic matter which it contained, yet this hypothesis is the one which accords best with the facts with which geology has latterly been enriched. In fact, we find no organized bodies belonging to what is called the primitive world in the strata of earths which modern geologists consider as the products of fire or of Vulcanism. They are only observed in the upper layers of the earth, in those of the latest formation, and in the soils which have evidently been precipitated in the midst of the waters. Aquatic animals existed before terrestrial animals. An argument which favors the hypothesis according to which the organic kingdom has been gradually developed and elevated from simple to more complicated forms, is drawn from the fact that we meet with remains of organic bodies belonging to the most simple species in the secondary and more ancient soils, whilst the most recent strata of the earth contain the remains of more complicated living bodies. The soils which rest directly on primitive rocks, present fragments of corals, radiated animals, and shells. It is only after these that remains of vertebrated animals, fishes, reptiles, and cetacea, are found in the water. Fossil bones of oviparous animals exist in the deep strata of the earth, whereas the viviparous mammiferæ are met with in the superficial layers. We observe the same in the organic complication of vegetables, whose remains are contained in the different layers of the earth. Impressions of cotyledonous plants, especially of ferns, are the first vegetable traces met with in the deep seated strata. Then come the remains of monocotyledonous plants, of arborescent gramina, of palms, etc., and finally those of the coniferæ and other dicotyledonous plants.

There have not yet been found any fossils belonging to apes or man, whose organization has reached the highest degree of complication and development. We may therefore admit, with great probability, that apes and men are the last and the newest products of our planet.*

Another circumstance favorable to the hypothesis of the gradual development of organic bodies, from the most simple to the most complicated, is that all those bodies, as well vegetables as animals, to this day appear in a simple form, at the period of generation, or when they proceed from the germ, and that it is only by degrees they acquire the most complex form peculiar to each species. To commence in a very simple manner, and to rise thence to the complicated, is the general character of every thing that has life, as well of individuals as of the entire of the organic kingdom.

These reasons, coupled with the fact that, after the extinction of the life of individuals, the materials of organized bodies are reduced to the most simple organic forms by the action of what is called spontaneous generation, oblige us to admit a primitive organic matter extended on the surface or in the crust and waters of our planet, concerning the first origin of which matter it is as possible for us to certify any thing as on that of the planet itself. This organic matter, with its different organic modifications, considered as matters of a peculiar species, sometimes is seen active and living in the individuals of vegetable and animal species actually existing, under conditions and in the midst of phenomena, the recital of which will be made hereafter; at other times remains merely capable of enjoying life, and endured with the faculty of taking on, in certain circumstances, the most simple organic forms, whenever it has been withdrawn from the composition of living bodies.

Several naturalists, particularly Buffon and Needham, have allowed the existence of a matter peculiar to living bodies. G. R. Treviranus concludes from his researches on life,

1. That there is in nature a matter which is ever mo ing, by which all living beings, from the byssus to the palm, and from the infusoria animalculæ to the sea monster, possess, life, and which, though immu-

* An additional argument in favor of this hypothesis, is the fact that whenever animal matter shall have lost that power which gave and maintained it in a higher degree of complication in form and functions—no matter how high this degree—it invariably returns to the most simple forms. The noble human form, after the cessation of the functions, possesses only sufficient plasticity to take on the shape of the lowest insects and worms. The same applies to the kingly lion of the forest and the soaring eagle. In fact, the matter composing each of these, after death, is in the same state as the matter which is described by Tiedemann as possessing merely the aptitude for life, and therefore taking on only the most simple form. Again, that external circumstances modify structure, is very well ascertained. The absence of light generally causes a mother to produce a deformed child, as Edwards observed in females confined in dungeons, whilst tadpoles, preserved from the light, became huge tadpoles instead of frogs. Natives of different climes have different parts of their organization prominently in action; the muscular system, for instance, is much more developed in cold than warm climates; on the other hand, natives of the tropics are from birth more excitab e than those of northern parts of the globe; in other words, the animal nervous apparatus is more developed. In a pure hypothesis it is not expected that the modus operandi of the circumstances to which Tiedemann alludes should be explained; collateral evidence is certainly in favor of it.—Trans.

table in its essence, is notwithstanding variable in its form, and is incessantly changing it.

2. That this matter is deprived of form in itself, but nevertheless ready to take that of life; that it maintains a determinate form under the influence of external causes; that it only continues in that form so long as these causes are active, and that it takes another so soon as new causes influence it.

3. That the matter capable of life, and the living principle, exist reciprocally, and that death is only a passage of certain forms of this matter to certain others."

Our author correctly observes that the various notions on this subject amount to nothing more than hypotheses, unsustained by proof sufficient to erect them into theories. It would be as easy for us to account, on natural or physical principles, for the origin of the various species of plants and animals as it would be to imagine how water sprang into existence without the creative fiat of some superior and superintending power. If we are therefore compelled to admit the existence of some unknown agency in the production of water, why not extend the same influence to the origin of the higher departments of nature? It is not necessary that we should admit that the whole was created at once. Each link in the mighty chain of creation may have been formed at periods more or less remote, and still man, with all his organs of thought and reflection, have been brought into being at last, as our author observes. Nay, this may not be all. The same infinite power may call another order of creation into existence, at the proper time, which will surpass man in intellectual endowments even farther than he does the animals now placed below him in the scale of life.

But we will leave this subject and pass on to some of the functions of the higher classes of animals. In relation to absorption, Tiedemann believes, from a comparison of this function in the various animals, that foreign matters gain access to the circulating fluid. It will also be seen from the quotation we are about to make that he is not a believer in the mechanical imbibition so much contended for by late physiologists.

"In all animals with naked skins, an absorption of liquid also takes place at the surface of this organ. The experiments of Leuwenhoeck, Baker, Fontana, and Spallanzani have proved that infusoria ratifera, vibriones, etc., absorb water. Polypi, medusa, radiaria, and worms, likewise, absorb rapidly to the skin. Entozoa, which live immersed in animal humors, absorb them by the skin, as Zeder and Rudolphi assert. Spallanzani found that snails absorb an abundance of water, for their weight increases rapidly when they are placed in it. Jacobson has lately made experiments on the absorbing power of the vine-snail (*helix pomatia.*) A solution of prussiate of potass, which was poured on the surface of animals belonging to this species, was absorbed with rapidity and passed into the mass of the blood. The blood can take up such a quantity, as afterwards to acquire a deep blue color when sulphate of iron is added. The absorption of water by frogs, toads, and salamanders, which takes place by the skin, and especially by that on the lower surface of the body, is shown by the valuable experiments of R. Townson, from which it appears these animals can absorb, by this medium, a quantity of water equal to the weight of their own bodies. Finally, Edwards has certified, by numerous experiments, that a very active absorption takes place by the skin in frogs, toads, and lizards. When these animals have lost much of their weight by a long exposure to the air, where their cuticular transpiration is very active, and are then immersed in water, so rapid an absorption of it goes on, that it very soon makes up the deficiency they had suffered. The absorption of water takes place more rapidly in warm than cold.

When we wish to account for the activity with which absorption is accomplished in animals, we meet with the same difficulty which impeded us when speaking of absorption in vegetables. Several physiologists, Magendie, Blainville, and Fodera regard absorption as the pure effect of capillarity, and to the cellular and also to the animal tissues they attribute the property of imbibing liquids, acting, as they conceive, in the manner of a sponge. Certainly the cellular tissue, the mucus and serous membranes, the common integuments, and according to the researches of Emmert and Lebkuchner, the walls of the vessels, possess the property of admitting fluids through them, or being permeable to liquids that are placed in contact with them, a subject to which we shall return in speaking of absorption in man; but this property does not constitute absorption, and only explains the penetration of the tissues. Absorption is also shown by the reception of fluids into certain spaces, namely, into the arteries in the four superior classes of animals, and by the impulse which is given to them in directions equally fixed.

The cellular tissue, as well as the mucus and serous membranes, possess the penetrability as much after death as during life; but this is not the case in the passage of the liquids. Besides, as we behold the reception and propulsion of fluids, during life, vary exceedingly according to the manifestations of activity, or of the life of animals, that absorption is more rapid in early than advanced age, and, finally, that divers influences and excitement are capable of modifying it, we are bound to consider it, as well as the propulsion of the liquids, as a vital phenomena, and we cannot reckon it among the purely mechanical effects of the capillarity of the tissues."

Our author does not agree with those writers who affirm that the saliva performs no part in digestion except mixing with, and moistening the food, so as to render deglution more easy and perfect. In order to give his opinion in relation to the parts performed by the various secretions in digestion we extract his remarks on that subject.

"Finally, the different humors, such as the spittle, the pancreatic juice, and the bile, which certain organs obtain from the nutritive fluid of animals, and mix with the food in different portions of the intestinal canal, are of the first importance in the act of assimilation.

In the first place, regarding saliva, this liquid is mixed in many animals with the aliment received into the mouth. Glands which secrete it are found in all the mammifera, (cetacea, probably, not excepted,) birds, and reptiles, among which last they are particularly large in serpents. Hither we must refer, also, the glands that secrete a poisonous humor in many ophidia, and which communicate with the inside of the poisonous fangs. In the class of fishes there are several that have glands analogous to the salivary ones. Of this kind, are, among others, according to Rathke, carps, eels, pikes, siluri, etc. Spallanzani also considered the liquid secreted by the internal surface of the pharynx in carps, barbels, and pikes, analagous to saliva. Among the mollusca, salivary glands exist in the cephalopoda, pteropoda, (clio,) and some gasteropoda (limax, helix, doris, aplysia, tritonia, onchidium, bulimns, murex, etc. Insects have organs in the shape of a sac, which secrete the spittle, and open into the buccal cavity. Ramdohr, Posselt, G. R. Treviranus, Rengerr, Leon Dufour, and others found these vessels in butterflies, bees, most diptera, libellula, cicada, many hemiptera, orthoptera, and some coleopter, in different aptera, (pulex,) lastly, in spiders, scorpions, and centipedes.

The saliva, a watery fluid, for the most part slightly alkaline, is composed, in the mammifera, of water, a peculiar animal matter called salivary matter, of mucus, ozmazome, perhaps a little albumen, and several salts. In some animals it also contains the sulpho-cyanuret of potassium. Its effects on the food are various. By the water and carbonates, acetate and hydrochlorate of potass and soda it contains, it assists in softening and dissolving the alimentary matters. It likewise destroys the phenomena of life in the food, which is especially the case in poisonous serpents, whose bite kills animals rapidly. Moreover it seems to favor the assimilation of the aliment by the azotized substances, the salivary and albuminous matters, which it adds to the food. In favor of its assimilating action, it may be alledged that animals which live on vegetables have larger salivary glands than those which feed on animal substances. Lastly, the addition of the saliva, by moistening and softening the food renders it more easy to be swallowed.

In many animals, when the aliment, now dissolved by the acid gastric juice, leaves the stomach and passes into the small intestine, two liquids of a particular kind are mixed with it, namely, the pancreatic juice and the bile. The former is secreted, in mammifera, birds, and amphibia, as also in rays and sharks among cartilaginous fishes, by a conglomerate gland, resembling the salivary, and called the pancreas. The simple, double or multiplex duct of this gland, sometimes opens directly into the first division of the small intestine; sometimes unites with the biliary ducts; occasionally one of its branches anastomoses with these ducts, whilst the other terminates in the intestine. In most bony fishes, the pancreas of the superior animals seems to be replaced by cul-de-sac appendages, more or less uumerous, of the small intestine, of which Swammerdamm gave the first exact description under the name of pyloric appendages (appendices pyloricæ.) and which in sturgeons (accipenser) are united and mixed into a mass resembling a gland. In regard to mollusca, Grant has lately perceived in some cephalopoda (loligo sagittaria) two glands of a bright red color and lobular form united to the biliary canal, which he thinks are analogous to the pancreas. He also considers the glandular appendages that communicate with the stomach in the aplysia and doris, as analagous to the pyloric appendages of fishes. The researches of Ramdohr inform us that among insects the intestinal canal is furnished, in many coleoptera, (carabus, cicindela, dytiscus, staphylinus, tenebrio, sylpha, nicrophorus, hister, and uttelabus) with cul-de-sac appendages similar to those of fishes.

The slightly acid pancreatic juice of mammifera, of the dog, the sheep, and horse. is composed, according to the researches I have made together with L. Gmeli, of water holding abundance of albumen in solution, a matter resembling casein, ozmazome, and different salts, whence it follows that there is no identity between it and saliva, as some physiologists have allowed. This liquid appears to be of use, chiefly by its richly azotized animal nature, which it imparts to the aliment dissolved in the stomach, in assimilating the food and bringing it into the condition of the animal chemical composition. It may be said in favor of this opinion, that the pancreas is much larger in mammifera and herbivorous birds than in carnivorous animals, and that if it be judged by its volume it supplies a more copious secretion. The pancreatic juice of birds, amphibia and cartilaginous fishes, has not yet been submitted to chemical analysis. The very abundant liquid contained in the pyloric appendages of fishes is whitish, viscous, mucilaginous, and mostly slightly turnsole reddens. We may presume that by its mixture with the chyme it also effects its assimilation.

The bile, the other fluid, which, poured in abundance into the intestinal canal, is mixed with the aliment after it has been dissolved in the stomach, is secreted by a large gland, having the name of liver, and remarkable for the peculiar arrangement of its blood vessels. It is found in all mammifera, birds, amphibia, and fishes, as also among the invertebrated animals, in all the mollusca and crustacea, in which latter it is often composed of a great number of branching canals. In the class of insects, it appears to be replaced by more or fewer vessels, ending in a cul-de-sac, which open into the intestinal canal, contain a yellowish liquid, of a bitter taste, and have been considered by Cuvier, Posselt, Ramdohr, Treviranus, Carus, and J. F. Meckel, as the secreting organs of the bile. Similar vessels are met with in aphrodita, in the class of annelida.

The bile of vertebrated animals is composed of water, mucus, and several peculiar animal matters, the resin of the bile, cholesterine, picromel, cholic acid, a coloring matter, and probably salivary matter, ozmazome, casein, and many salts. In these animals it is chiefly taken from venous blood, carried to the liver by a large venous trunk, the vena portæ, which is distributed within this gland like an artery. Its secretion seems designed, on one hand, to keep the mass of the blood in such a state, in regard to chemical composition, as to enable it to operate in nutrition, and, on the other hand, to co-operate in the accomplishment of the assimilating act of the alimentary matter.

The majority of the materials of the bile, the resin, fat, coloring principle, mucus, and salts, are ejected with the undigested remnants of alimentary matters, in conjunction with which they constitute the excrements. From this it appears then, that this secretion has a design relative to the maintenance of the chemical composition of the blood. As to the part it acts in digestion, it not only consists in exciting, by its bitter resin the mucus membrane of the alimentary sac to furnish a more abundant secretion, and the muscular tunic to perform more vehement movements, but also, by the azotized principles it contains, as picromel, ozmazome, and cholic acid united with the dissolved aliment, in assimilating and reducing it to the animal chemical composition. What speaks for the re-absorption of these principles, with the dissolved alimentary substances, is that they are not discovered in the excrements.

The changes the aliments undergo in the alimentary sac, in order to become fit to be absorbed, consist, therefore, in general, in the destruction of qualities which they may yet preserve from their organic origin, and in the communication of other properties, which make them capable of becoming constituent parts of the body of the animal that takes them. If they are yet living and organized, their life and organization are destroyed. Moreover, the chemical composition, which had been given them by other living bodies, changes, and they are resolved into their simple organic elements. Finally, they are converted into a liquid resembling in chemical composition the mass of the liquids of the animal that has received them; this liquid is called chyle, and is absorbed. This change is effected partly by movements, and partly by the addition of secreted liquids. An ejection of excrement, which takes place in most animals, is the final limit or consequence of this series of operations. The manifestations of activity or life which cause these changes in alimentary substances are designated by the name of digestion or assimilation in the first passages.

No proper digestion, division, solution, and fluidification of solid aliment, is observed in plants, which can only take up liquid food by absorption. There appears to be in them nothing more than assimilation of these alimentary liquids by the juices that mingle with them. Neither do plants give out excrement. Thus digestion is designed to take from aliments the particular qualities that have been communicated to them by other living bodies, and to impress new ones upon them which allow of their becoming constituent parts of the nutritive juice and solid parts of the being that feeds on them.

In animals furnished with a lymphatic system to receive and convey the chyle, the nutritive juice continues to be elaborated whilst passing through this system. Secreted fluids are joined to the chyle and to the lymph of the blood, which bring them more and more near to the condition of the blood. Among the organs which act in the secretion of these liquids, we reckon glands of a particular kind, the lymphatic, as also the spleen, the supra-renal capsules, and the thyroid gland. The cyle and lymph approach the nearer to the blood in regard to color, coagulability, and constitutent principles in proportion as assimilative liquids are added to them, as I shall hereafter show when speaking of the assimilation of absorbed substances in the lymphatic system of man. These glands, which, in fishes and amphibia, appear with the lymphatic system itself, increase in number and volume, in birds and mammifera, whose more intense manifestations of activity, require a more rapid preparation of blood for their support."

As many of our readers may not have examined the subject of nutrition to any extent, in plants, we will present them with a few extracts from our author on it. Some contend that plants possess the power of forming many of the ultimate constituents of which they are composed from the elements with which they are surrounded. Not long since we heard a learned and eloquent professor declare that they could *manafacture*, or rather *create*, even carbon, a substance which has been always ranked among the simple bodies. Tiedemann is decidedly opposed to this doctrine. He declares that plants and even animals are nourished in no other way than by the arrangement of the different elements presented to them. They cannot create the carbon, calcium, or iron found in their structures. This doctrine alone appears to us tenable. But we will let our author speak for himself on the subject. After presenting a variety of arguments he comes to the following conclusions.

"If we compress into a small space the phenomena of the respiration of plants, we see that they consist in the exhalation of water in the vaporous form, and of oxygen gas during the day, under the influence of the sun's light. The water comes from the sap which the roots send, by the sap vessels, into the parenchyma of the leaves, and from the moisture these absorb in the night-time. The primary effect of the evaporation of this water is the condensation of the organic matters contained in the sap. The oxygen gas is in great part taken from the carbonic acid absorbed during the day, and from the water impregnated with this gas, which the roots bring from the soil, together with the organic matters; these are the sources to which Senebier, Woodhouse, and Saussure assign it. Possibly, it also partly arises from the oxigenated organic combinations contained in the sap, that is, from the acetic acid, the sugar and the matter analagous to gum. It is not certain that the water of the sap is decomposed by the respiration of plants, as Berthollet and Thomson have supposed, and that a part of the oxygen proceeds thence; Saussure rejects this decomposition as by no means probable. The exhalation of oxygen increases the proportion of carbon relatively to the other elements of the sap, just as its absolute quantity becomes greater by the absorption of the carbon contained in the carbonic acid of the atmosphere. In support of this hypothesis, the experiments of Chaptal, Hassenfratz, and Senebier, may be quoted, from which it follows, that plants which have increased in the shade contain much less carbon, than others which have been exposed to the light.

The reception and decomposition of carbonic acid by the influence of the sun, as well as the absorption of oxygen gas and the production of carbonic acid during the night, are to be considered as so many organic actions of living leaves, which take place in the substance of these organs. These actions continue so long as the leaves are fresh and perfect, and even after they have been cut into pieces. But when the leaves are crushed, so as to destroy their organization and life, carbonic acid gas is no longer decomposed under the influence of the solar light, neither does the absorption of oxygen in darkness any longer take place. The vegetable mass then only converts a small quantity of the oxygen of the air into carbonic acid, as dead organic matters do.

The acts of respiration which living leaves execute under the influence of light, are of the highest importance to the life of plants. If plants be deprived of their leaves, or if they lose them by cold or the destructiveness of insects, their nutrition and growth are stopped, the development of the flowers, the act of fecundation of the fruit and seeds cease to occur, and the already formed fruit does not ripen. It is true, that perennial plants then shoot out new leaves, because the buds which should not open until the year after are developed, but this loss does not on this account less frequently cause the death of vegetables.

If we inquire in what respect respiration is necessary to the life of plants, we can find no other use for it than to produce the nutritive juice, properly so called, or the cambium, from the sap sucked by the roots. The sap, which reaches the leaves colorless, not coagulable, without globules, and composed of water holding in solution carbonic and acetic acids, a gummy-saccharine matter and divers salts, is in them converted into a greenish liquid, partly coagulable and filled with globules, which the nutritive vessels return into the trunk of the plant, where it serves for the proper nutrition, as also to the formation, the development and growth of the parts. It is from this liquid that in perennial plants, the matter necessary to the production of new ligneous and cortical layers is deposited; it is this which furnishes the materials of which new shoots are formed.

The juice expressed from the leaves contains the green fecula, which is precipitated as a sediment. In this fecula are perceived green grains or globules, which do not exist in the sap. From the experiments of Rouelle, Einhof, Proust, Vauquelin, Pelletier, and Caventou, it follows that it is composed of a green resinous matter, soluble in alcohol and ether, and combustible, called chlorophylle, of starch, or matter like gluten, and vegetable albumen. When the juice is heated, partly coagulates in flakes, and acids precipitate it. Senebier and

Gough clearly demonstrated that the green color of plants depends on respiration, subservient to the influence of light. The conversion of the matters contained in the sap, of the carbonic acid suspended in water, of the acetic acid, of the sugar, and the gum, into more compound organic combinations, such as they exist in the green fecula, are to be considered as an effect of respiration for which no satisfactory theory has yet been given. From the facts hitherto obtained concerning respiration, the following is the least strained explanation of it. The matters existing in the sap, the acetic acid, and particularly the gummy saccharine principle, are organic combinations of an inferior kind, containing a vast quantity of oxygen relative to the carbon. Again, in the fecula are found starch, a substance approaching to gluten, and albumen, in the composition of which matters less oxygen enters in proportion to the carbon. It is precisely these changes in the respective proportions of the two elements, which seems to be the result of respiration, since the absorption of carbonic acid from the air augments the mass of carbon, either absolutely or relatively to the oxygen, and the quantity of the latter is perhaps diminished by exhalation. From this it follows, that the organic combinations of a lower degree, that exist in the sap, are converted into others of a higher degree, which are found in the green fecula. Lastly, in regard to the azote contained in the glutinous matter and albumen of this fecula, it is probably taken from the humors, and is already existing in the sap, wherein some chemists have found an azotized substance. With the formation of organic combinations of a higher degree which accompanies respiration, seems also to be connected the first appearance of the organic materials of aggregation or globules.

The nutritive juice which is prepared in the leaves, by the act of respiration, under the united influence of light and heat, and which contains organic combinations of a superior kind, starch, resinous matters, albumen, and gluten, is the liquid which particular vessels carry out of the leaves and transport to the different parts of the vegetable, to serve for their nutrition. We shall return to this point when speaking of the circulation of the nutritive juice and nutrition. In other respects the nature of the cambium appears to vary according to the species, as may be imagined from the differences remarked in the composition of vegetables and their products. Notwithstanding the resemblance of the alimentary matters imbibed by the roots, and the equality of the external influences which maintain respiration, a phenomenon of which chemistry has hitherto been unable to give an explanation, however imperfectly satisfactory, its diversity can only be considered as an effect of the plastic activity which is manifested in a special manner in the different species of plants, and by virtue of which each vegetable species prepares a nutritive juice adapted to its necessities.

To come to an end, we shall just glance over the changes produced in the atmospheric air by the roots, the flowers, and the fruit. It is proved that these organs do not occasion the same changes as those effected by the green parts of vegetables, especially by the leaves. Roots that have recently been dug from the soil, when placed in a receiver full of moist atmospheric air, from which the stalk and flowers project, and the ends of which alone are immersed in water, absorb oxygen and exhale a little carbonic acid gas during the day, according to the experiments of Th. de Saussure. They therefore act like the leaves during the night. When he introduced nitrogen, hydrogen, or carbonic acid gas into the receiver containing the roots, the plants soon perished.

The action of the flowers on the atmosphere likewise differs from that which the leaves exert. Th. de Saussure found, in his experiments, that all, even those of aquatic plants, absorb oxygen gas, and that they are not developed in media deprived of this gas. They faded in a vacuum and in nitrogen gas. When a flower is placed under a receiver full of atmospheric air, and stopped by a bath of quicksilver, the quantity of air is but slightly diminished, or even is not at all diminished so long as there remains any oxygen gas. The flower absorbs the oxygen and replaces it by a nearly equal quantity of carbonic acid gas. The operation is accelerated by the influence of solar light and heat, whereas it is much more slow in the shade. In general, equal weights of flowers produce more carbonic acid gas than green leaves disengage in darkness in the same space of time. The absorption of oxygen gas, and the production of carbonic acid gas, take place chiefly by the genital organs. Formerly Saussure supposed, and Grischow also thought he remarked, that flowers exhale nitrogen. But in his later experiments, he was convinced they gave out neither azotic nor hydrogen gas.

Regarding the changes which the fruit occasions in the atmospheric air, Th. de Saussure found green fruit determine similar ones to those produced by the leaves. Exposed to the air, they absorb, according to him, carbonic acid gas and exhale oxygen, in smaller quantity, however, and less freely as they approach the period of ripeness. Berard, again, assures us, that he remarked, in his experiments on the ripening of fruit, that green fruits, raspberries, pears, apples, apricots, figs, cherries, gooseberries, grapes, etc., do not act like the leaves at any period of their growth, under the influence of solar light; that they do not absorb any carbonic acid gas, nor do they exhale oxygen. He maintains that their sole action on the atmosphere, as well in light as shade, consists in the absorption of oxygen and the exhalation of carbonic acid. This contradiction determined Saussure to undertake fresh experiments, and he has shown that green fruits, cherries, plums, pears, and grapes, disengage oxygen and absorb carbonic acid, in the solar light, as well in air containing carbonic acid as in water holding this acid; that, on the other hand, in darkness, they absorb oxygen and exhale carbonic acid gas, and that consequently they act on the air in the same manner as the leaves, although in a less powerful degree. If their growth go on tardily, they destroy the purity of the air under any circumstances, but less in light than in darkness. Lastly, he thought he found that, in the immature state, and at the time they began to get sour, they also absorb a part of the oxygen of the air, which might consequently contribute to the development of their acidity."

We have quoted largely on this subject from the pages of our author, because vegetable physiolgy, a most interesting subject, is not frequently examined by our authors. It will readily be seen that vegetables and animals harmonize with each other in the great plan of nature. The carbon thrown off from the lungs of animals, in the process of respiration, is immediately consumed by vegetables, which in turn furnish the oxygen necessary for animal nutrition.

The evolution of heat in animals has been the source of much controversy among physiologists. Some suppose that it is a secretion, some that it is the offspring of the friction which arises from the passage of the molecules composing the fluids, in the different currents of the circulation, while others affirm that it depends entirely upon chemical phenomena. It seems to us that we have much to learn in relation to the cause of heat in animals. Unless we admit that it is a compound substance, formed by the union of different elementary bodies, it cannot be a secretion. Secretory organs can do nothing more than separate and combine the various elements presented to them by the circulation. Until heat is proved to be either a compound *body*, or is found, as an elementary substance, floating in the fluids of the body, it cannot be said to be secretion. Objections equally strong may be brought against the remaining hypotheses which have been urged upon the notice of the profession for the last century. The truth is that we are in the dark on the subject, and we will remain so unless some future physiologist should be more successful in his investigations than his predecessors. The calorific process in animals is undoubtedly the result of nutrition but in what way it is accomplished is unknown. The following are the remarks of Tiedemann on this interesting portion of physiology.

"With respect to the cause of the production of heat in animals, there are few vital phenomena on which so many different theories have been built as this. The iatro-mechanicans taught that heat was produced in living bodies as in those which possess no life, by the friction which takes place both between the fluids and the coats of the vessels, and in the organs themselves, in consequence of their internal movements. The physicians of the ancient introchemical school regarded it as a result of the mixture of the supposed acid chyle with the alkaline blood, which they said caused an effervescence, accompanied by an evolution of heat. The partisans of the new chemical doctrines believe the cause of animal heat to be found in the act of respiration, which they compare to a combustion going on between the materials of the venous blood and the oxygen of the inspired air, in whom the caloric evolved combines with the arterial blood, and is distributed over the body. Other physiologists seek the source of it in digestion, in nutrition, secretion, or even in the nervous system. Without stopping to examine these theories, of which we shall treat more fully when at the production of heat in man, we shall merely mention here what is certain, namely, that none of them offer a satisfactory explanation. Even the doctrine of Crawford and Lavosier, according to which heat is a product of respiration, although it has the suffrages of most naturalists, has been found insufficient by Dulong and Despretz, who endeavored to ascertain by experiment how far the quantity of oxygen gas consumed in respiration, sufficed to produce all the heat which animals are continually losing, and who, after this, were induced to allow other sources of heat, which are unknown to us.

The only point that can be regarded as placed beyond doubt is, that the evolution of heat is a vital act which depends immediately on the process of nutrition, the conditional and preservative cause of life. The taking of alimentary matters and their assimilation by digestion and respiration, the circulation of fluids, nutrition, and secretion, the renewal of materials that accompanies the exercise of life, and the incessant changes of composition in the solids and liquids, all which are under the influence of the nerves, also act a part in the production of heat, and it is erroneous to seek the cause of it in any one of these acts only. The intensity of the evolution of heat and the property of maintaining itself at a certain temperature proper to each species, are in animals, in direct ratio with the composition of their organization, and with the sum and intensity of their manifestations of activity. Birds and mammifera, which take aliment at the shortest intervals, which digest with the greatest rapidity, which consume the most oxygen, and give out the most carbonic acid, whose circulation is most rapid and energetic, which exhibit the greatest pertinacity in their movements, in which we observe the strongest effects of the nervous system, which secrete the greatest quantity of diversified humors, and in which, in short, all the phenomena proclaim that the renewal of matters takes place in the most speedy manner; these have the highest degree of heat, and are able to maintain it with the greatest uniformity at the temperature proper to each of them. Amphibia, fishes, insects, mollusca, and worms, whose structure is less complex, whose vital phenomena exhibit less diversity, and in which the above-named actions of life have less intensity, have also a lower degree of heat, are more subject to variation in their temperature, and have their faculty of generating caloric confined to smaller limits.

Further, the generation of heat resulting from the renewal of matter, and the changes of composition ever accompanying life, varies in animals, within certain limits, according to the development, the periods of age, the nature of aliment, and the manner of performing digestion, according to the respiration, the circulation of the blood and the nervous influence, the seasons, even according to the periods of the day, during waking and sleeping, according to the external stimuli that affect animals, and finally, according to diseases, medicinal remedies, and poisons. The proofs in support of this assertion shall be given when we treat of the heat of man.

In plants, an evolution of heat seems to occur, though only to a small degree, during the acts of respiration, nutrition, and secretion, as also during fecundation and germination. But vegetables do not appear to undergo continual changes in consequence of their internal activity, in their solids when once formed, as is observed in animals, in which the matter of the different tissues is incessantly changing; neither do they execute voluntary movements, nor does the entire group of nervous functions belong to them, so that they lack the chief sources of the generation of heat."

Our author believes life to be the result of external forces operating upon the excitability of living bodies. He affirms that this excitability is a property inherent, not only in all organized beings but in all their parts. It differs, however, not only in animals, and plants but also in all the organs and tissues of which they are composed. Each vegetable and animal species lives in a medium, and under circumstances peculiar to itself, and the whole is governed by certain fixed laws applicable to the entire mass. As this subject forms the basis of the work we will let our author speak for himself upon it.

"From these inquiries it follows, that external influences or excitements that urge living bodies to action, whether of the inorganic or organic kingdom, are the cause of their preservation and their persistence in the state of life, but do not produce life itself. Their action supposes the previous existence of an organic activity which they only excite and determine to action. That an external should act as an excitant on an organized body, and urge it to a development of power, the body must possess the property and capability of being affected by external things, and of acting by a proper force, whenever their influence is exerted over it. The excitement produced by an external object in a living body is not, therefore, a passive state but an active one, is an act of life, which supposes the existence of organic forces. Inasmuch as these forces are diversified in living bodies, insomuch are the effects of excitants varied. This throws light on the fact, that the phenomena of life are manifested in a specific and uniform manner in each organized being, notwithstanding the diversity of excitements, and that different living bodies develope a different activity in a similarity of external things.

Excitability, or the faculty of being affected by excitants and placed by them in a state of excitement is communicated, like the plastic force, to the germ of organized bodies, by the activity of the generator organisms, which produce the specific constitution of their organic materials, and the quantitative division of these materials.

It is manifested in each germ in a specific manner corresponding to the species of the generator bodies, and under the influence of fixed excitants. The plastic force of germs endued with excitability gradually produces all the tissues and organs, together with their different organic or vital properties, observing, at the same time, the track of formation and development of the species, and in concurrence with the excitants, which induce it to become active. Subsequently we see other manifestations of force developed, according to the varied nature of the organs that have been produced by formation.

Regarding the dependence of organized bodies on external agents, it is founded directly upon the fact, that the latter furnish both the stimulus and the materials by which the acts of formation and nutrition, of which the existence and preservation of living bodies is the consequence, are executed. Heat causes the plastic force inherent in vegetable and animal germs to enter into action, urges it to formation, and places the organic matter of the germ and the ovum, with which it combines, in the condition essential to development. No germ is developed without a certain degree of heat. Its influence is necessary to the continuance of the operation of nutrition of all vegetables and animals, because the assimilations of aliments, respiration, the acts of nutrition in the solid parts, and the secretion of fluids, are accompanied with organic changes in the composition, which can only take place at certain temperatures. All the other manifestations of force of living bodies, movement, sensation, generation, are only effected at determinate temperatures, as I shall afterwards show under the head of heat, as an external condition of life. The stimulus of light also in plants, and even in most animals, is an important agent for the maintenance of the acts of formation, and for the excitement of all the acts of life.

The other externals on which the maintenance of organized bodies depends, such as atmospheric air, water, and aliments, act as excitants to parts with which they are placed in contact, and from these matters it is that living bodies, in the act of assimilation and respiration, prepare the formative and nutritive fluid by the activity peculiar to them. This fluid is absolutely necessary to the preservation of the activity of the organisms, inasmuch as being an excitant, it determines the action of the solids, and furnishes the materials by which they are maintained, by the act of nutrition, in possession of their organization and vital properties. Hence, the necessity of these external agents, for the continuance of organized bodies and the maintenance of their activity, will appear. Without them, no vital phenomena, no action of the organic powers, are developed. Withdrawn from their influence, the life of organized bodies is infallibly extinguished.

Besides the external agents necessary to the presevation of organized bodies, as heat, light, air, water, and aliments, there are moreover internal excitants inherent in living bodies, which themselves produce, and which maintain them in activity. Of these are the exciting fluids of all vegetables and animals which are the products of their own activity. In the fecundated matter of the germ of each plant and animal, a liquid, containing globules that move under the influence of heat is formed. Solid parts proceed from the constituent principles of this fluid, and take an organic texture. In the vegetable and animal embryo, during development, organs are formed which by the vital properties imparted to them in the formative act, take up by absorption the alimentary matter furnished by the parent body to the grain or ovum, and convert it to their own nutritive fluid or blood, by the acts of assimilation and respiration. Spaces are marked out in which the formative liquid flows, and this furnishes the stimulus that urges the contractile parietes of the spaces to perform movements. Thus it is, that in embryons that are gradually, rapidly, or slowly developed at fixed periods, and in determinate order, organs are produced from the formative fluid, which themselves elaborate, which, from their vital properties, are enabled, under the exciting influence of the formative liquid, to fulfil the fractions essential to the preservation of the newly organized being."

The remaining pages of the work we have noticed thus far, consists in remarks on organic contractility with continued references to the early parts of the book. Although we do not agree with our author in all his doctrines, still as a whole the work is a valuable addition to our physiological libraries. W. W.

